# Small but mighty: the power of microcrystals in structural biology

**DOI:** 10.1107/S2052252525001484

**Published:** 2025-03-13

**Authors:** Courtney J. Tremlett, Jack Stubbs, William S. Stuart, Patrick D. Shaw Stewart, Jonathan West, Allen M. Orville, Ivo Tews, Nicholas J. Harmer

**Affiliations:** a Living Systems Institute University of Exeter Stocker Road ExeterEX4 4QD United Kingdom; b Department of Biosciences University of Exeter Stocker Road ExeterEX4 4QD United Kingdom; c School of Biological Sciences, Faculty of Environmental and Life Sciences University of Southampton SouthamptonSO17 1BJ United Kingdom; d Diamond Light Source (United Kingdom) Harwell Science and Innovation Campus DidcotOX11 0DE United Kingdom; e Defence Science and Technology Laboratory Porton Down SalisburySP4 0JQ United Kingdom; f Douglas Instruments Ltd, East Garston, HungerfordRG17 7HD, United Kingdom; g Institute for Life Sciences University of Southampton SouthamptonSO17 1BJ United Kingdom; h Cancer Sciences, Faculty of Medicine University of Southampton SouthamptonSO17 1BJ United Kingdom; i Research Complex at Harwell Harwell Science and Innovation Campus DidcotOX11 0FA United Kingdom; Uppsala University, Sweden

**Keywords:** microcrystals, MicroED, serial crystallography, time-resolved studies, seeding, phase diagrams, sample preparation, sample delivery

## Abstract

Advancements in macromolecular crystallography, driven by improved sources and cryocooling techniques, have enabled the use of increasingly smaller crystals for structure determination, with microfocus beamlines now widely accessible. Initially developed for challenging samples, these techniques have culminated in advanced beamlines such as VMXm. Here, an *in vacuo* sample environment improves the signal-to-noise ratio in X-ray diffraction experiments, and thus enables the use of submicrometre crystals. The advancement of techniques such as microcrystal electron diffraction (MicroED) for atomic-level insights into charged states and hydrogen positions, along with room-temperature crystallography to observe physiological states via serial crystallography, has driven a resurgence in the use of microcrystals. Reproducibly preparing small crystals, especially from samples that typically yield larger crystals, requires considerable effort, as no one singular approach guarantees optimal crystals for every technique. This review discusses methods for generating such small crystals, including mechanical crushing and batch crystallization with seeding, and evaluates their compatibility with microcrystal data-collection modalities. Additionally, we examine sample-delivery methods, which are crucial for selecting appropriate crystallization strategies. Establishing reliable protocols for sample preparation and delivery opens new avenues for macromolecular crystallography, particularly in the rapidly progressing field of time-resolved crystallography.

## Abstract 2

Developments in macromolecular crystallography now allow the use of microcrystals for structural analysis through advanced beamlines and techniques such as microcrystal electron diffraction and room-temperature crystallography. This review addresses methods of matching microcrystal preparation and sample delivery. The use of microcrystals enhances the possibilities in fields such as time-resolved crystallography.

## 1. Introduction

Deciding on the optimal modality to address a biological question and prepare the corresponding sample is a challenge, with guidance scattered throughout the literature and across user experiences. In this review, we compile the resources to help resolve these choices and inform the experimental design to obtain static or time-resolved structures from microcrystals.

Microcrystals have played a pivotal role in developing microfocus beamlines at synchrotron facilities. Pioneering studies using microbeams at ID13 (Cusack *et al.*, 1998 ▸) at the European Synchrotron Radiation Facility (ESRF) paved the way for the construction of ID23-2, the first dedicated macromolecular microfocus beamline (Flot *et al.*, 2010 ▸). Today a growing number of microfocus beamlines are available, where data collection from microcrystals is possible, and an overview of these facilities has been presented in a recent review by Chavas *et al.* (2024 ▸).

**Figure 1:**
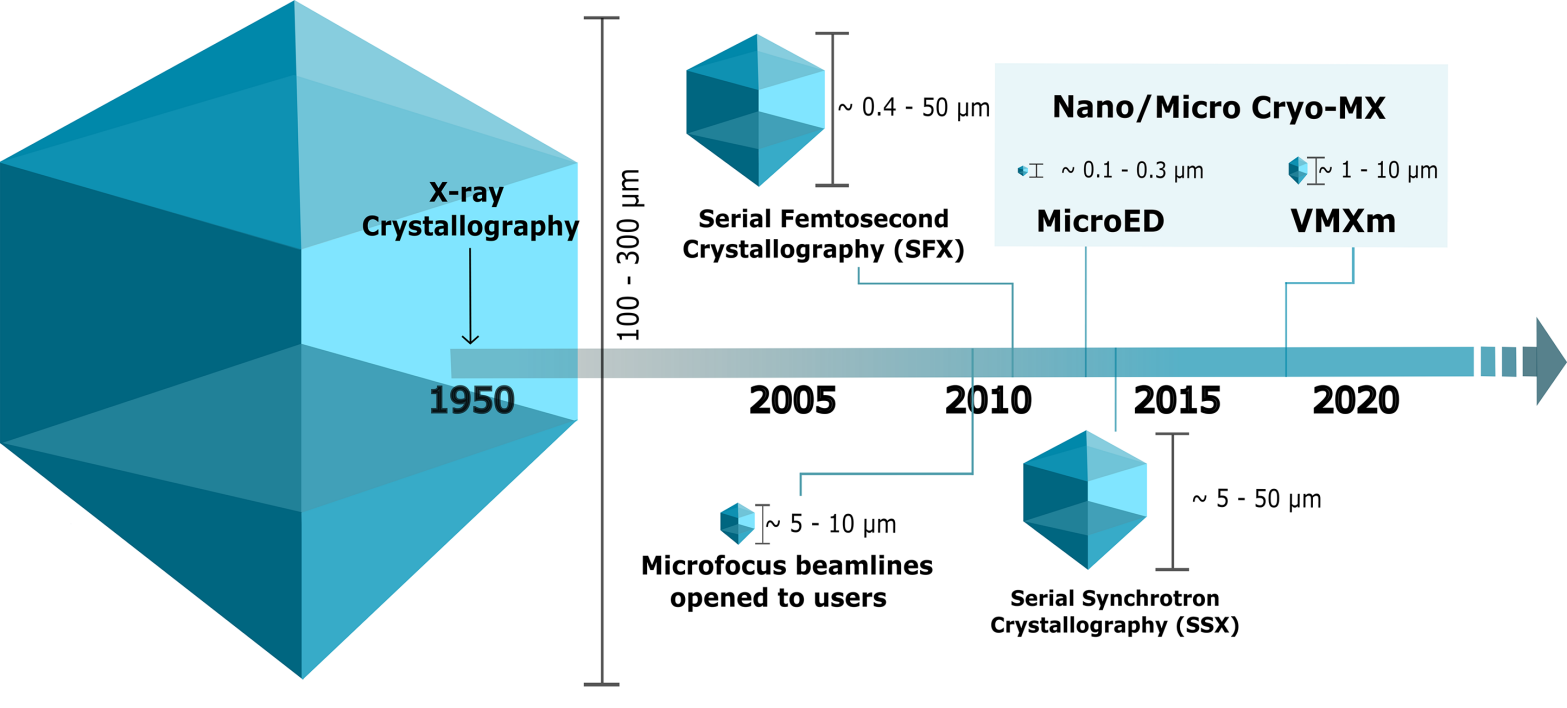
The evolution of macromolecular crystallography, told by size and method. Macromolecular X-ray crystallography (MX) gave rise to the first 3D protein structure, of myoglobin, determined by Kendrew and coworkers in 1957 (Kendrew *et al.*, 1958 ▸). Crystals for MX structure determination were required to have sizes greater than 100 µm in all dimensions to expose sufficient crystal lattice volumes to the beam (Smyth & Martin, 2000 ▸). Study of 5–10 µm crystals was made possible by the development of advanced instrumentation with dedicated microfocus beamlines such as ID23-2 at ESRF (Flot *et al.*, 2010 ▸). The advent of single-shot serial crystallography at XFELs opened up the opportunity to study samples (and/or reactions) at physiological temperatures. The first structure reported from this method was lysozyme (Boutet *et al.*, 2012 ▸). While serial methods were adopted by synchrotron sources, crystal sizes are typically larger and range between 10 and 50 µm, as seen for the first structure determined by this method (Gati *et al.*, 2014 ▸). Sample preparation using methods adapted from cryoEM include MicroED on one hand, with the first published structure in 2013 (Shi *et al.*, 2013 ▸), and X-ray crystallography on the other hand, with the VMXm beamline at the Diamond Light Source (Warren *et al.*, 2024 ▸), which are designed for the use of samples down to submicrometre sizes. Despite the plethora of methods, macromolecular crystallization currently remains a bottleneck for structural biologists.

Microcrystals have been applied to room-temperature serial femtosecond crystallography (SFX) experiments at X-ray free-electron lasers (XFELs; Chapman *et al.*, 2011 ▸, 2014 ▸; Barends *et al.*, 2022 ▸). The first high-resolution structure from a slurry of microcrystals (1 × 1 × 3 µm) was published in 2012 (Boutet *et al.*, 2012 ▸). A second important development is crystal diffraction in cryo-electron microscopy (cryoEM), where appropriate sample preparation now enables data collection from submicrometre crystals/nanocrystals (micro/nano cryo-MX). A key application in this area is microcrystal electron diffraction (MicroED), where tilt-series data sets are acquired with minimal sample volumes (Shi *et al.*, 2013 ▸). Collectively, these methods have reduced the size of the crystal sizes that can be effectively studied by structural biologists from nanometre to micrometre scales (Fig. 1 ▸).

## 2. Microcrystal electron diffraction (MicroED)

MicroED uses a transmission electron microscope (TEM) to collect data sets from crystals with depths restricted to between 100 and 300 nm in all dimensions to reduce the possibility of multiple diffraction events from the lattice planes (Nannenga & Gonen, 2019 ▸; Martynowycz *et al.*, 2021 ▸). There are now ∼150 structures in the PDB determined by MicroED (as of 31 October 2024). Of these, several could not be obtained by other methods, including some membrane proteins (Zhu *et al.*, 2020 ▸; Gallenito & Gonen, 2022 ▸; Martynowycz *et al.*, 2023 ▸), hard-to-crystallize samples (Haymaker *et al.*, 2023 ▸) and radiation-sensitive samples (Martynowycz *et al.*, 2020 ▸). For crystals between 1 and 10 µm in one dimension, advanced nanofocus X-ray beamlines can be used to collect diffraction data utilizing the MicroED sample workflow (Beale, Waterman *et al.*, 2020 ▸; Crawshaw *et al.*, 2021 ▸; Warren *et al.*, 2024 ▸).

Electrons interact more strongly with biological matter compared with X-rays (Henderson, 1995 ▸), producing multiple diffraction planes and thus more comprehensive information per image through rotations. The considerably smaller electron wavelength at typical electron-microscope voltages leads to a larger Ewald sphere in comparison to X-rays, intersecting with more points in reciprocal space at a given time. However, this also results in the collected diffraction image lacking unit-cell information, so data from multiple crystals are merged and processed (Clabbers & Xu, 2021 ▸). Due to these characteristics, higher resolution structural information can be collected from smaller crystals with lower doses in multiple short exposures, providing higher signal to noise compared with X-ray methods (Henderson, 1992 ▸). Additionally, electrostatic potential maps are influenced by core and valence electron densities, allowing the bonding environment and oxidation states to be observed. However, this high interaction imposes an upper limit on MicroED crystal size to crystals thinner than twice the mean free path of the incident electrons to limit multiple elastic scattering events. When this increases, the diffraction pattern will less reliably follow the expected structure-factor relationship, making data processing more challenging (Martynowycz *et al.*, 2021 ▸; Drevon *et al.*, 2023 ▸).

Improvements in sample preparation and continuous rotation data collection led to a 0.87 Å resolution structure of lysozyme, demonstrating the current capabilities of the technology and methodology (Martynowycz *et al.*, 2022 ▸). MicroED provides better definition of hydrogen atoms compared with X-ray data of similar resolution due to the improved contrast of hydrogens next to heavier atoms (Clabbers *et al.*, 2022 ▸). Most MicroED experiments are performed at 200–300 keV. However, ultra-low dose collections can routinely produce structures with resolutions below 3 Å (Lanza *et al.*, 2019 ▸). The TEM is partnered with one of two detector types: a direct electron detector (DED) or an indirect electron detector. DEDs provided a generational improvement on older detector technology (charge-coupled device detectors) by eliminating the requirement for light conversion and scintillation. These steps previously resulted in considerable background noise. The newest technology in DEDs offers higher precision by counting the electrons detected and offering advanced filtering to generate higher signal to noise. Detectors using electron counting offer higher resolution structures at lower doses from smaller crystals and minimize radiation damage (Martynowycz *et al.*, 2022 ▸), a feature that can be crucial when studying radiation-sensitive proteins. Electron counting mode is now the most widely used for MicroED, but many detectors also offer an integrating mode which can be used for less radiation-sensitive samples and faster data collection.

On the X-ray side, VMXm is an advanced nanofocus beamline that produces a stable 0.3 × 2.3 µm (vertical × horizontal) X-ray beam for diffraction data collection that is coupled with a very low sphere-of-confusion goniometer for sample rotation (Warren *et al.*, 2024 ▸). The tuneable X-ray energy (6–28 keV) allows data sets to be obtained at higher energy, prolonging the crystal lifetime in the beam using smaller samples, which together lead to gains from photoelectron escape (Storm *et al.*, 2020 ▸).

Both MicroED and data collection at VMXm are performed *in vacuo* to reduce noise as scattering from air. Additionally, the cryogenic conditions used for electron and X-ray diffraction help to reduce the effects of X-ray-induced photoelectric effects commonly referred to as ‘radiation damage’ (Henderson, 1995 ▸; Beale, Warren *et al.*, 2020 ▸; Warren *et al.*, 2024 ▸). Electron beams, like X-rays, cause ionizing radiation damage in biological macromolecules, posing challenges for MicroED studies. Evidence shows both global and site-specific radiation damage, with the average intensity of all reflections in proteinase K nanocrystals decreasing by 73% after 1 e^−^ Å^−2^ exposure (Hattne *et al.*, 2018 ▸). This fluence corresponds to a dose of 4.5 MGy, which is much lower than the Garman limit of 30 MGy for X-ray cryo-MX, likely due to resolution differences (Baker & Rubinstein, 2010 ▸). Site-specific electron radiation damage has been observed below 1 e^−^ Å^−2^ at sensitive sites, including metal centres, disulfide bonds and acidic residues, mirroring X-ray cryo-MX findings (Hattne *et al.*, 2018 ▸). Fractionating the dose through serial approaches reduces radiation damage, akin to serial synchrotron crystallography (SSX) and serial femtosecond crystallography (SFX) (Bücker *et al.*, 2020 ▸). While MicroED and single-particle cryoEM typically report dose in e^−^ Å^−2^, expressing it in grays would enable systematic comparisons across techniques. The *RADDOSE-3D* GUI now facilitates the conversion from ‘fluence weighted dose’ to ‘diffraction-decay weighted dose’, enabling doses to be quoted in grays (Dickerson *et al.*, 2024 ▸).

In both MicroED and data collection at VMXm, crystals are pipetted onto one side of a glow-discharged carbon-coated grid, blotted to remove excess liquid in a humidity-controlled environment and then vitrified by rapidly plunging them into liquid ethane (Nannenga & Gonen, 2014 ▸). Reducing excess liquid around crystals and matching the sample to the beam size results in reduced background, thereby improving data quality (Warren *et al.*, 2015 ▸, 2024 ▸). Usually, data from fewer than ten deposited crystals on the grid are required to obtain a complete data set both for MicroED and at VMXm; however, settling on the grid can limit crystal lattice orientations, which consequently leads to missing cusps of the Ewald sphere. Ideally, crystals would fall randomly across the grid surface, providing access to all angles of rotation. However, this is often not the case with fixed methods and is particularly tricky with a plate-like crystal morphology. Frequently these crystals will lie on a preferred axis that has more surface-area contact with the grid (Gillman *et al.*, 2024 ▸). This can make a minimal impact on the data completeness if the crystal has high symmetry. For example, a crystal with a high-symmetry point group, such as cubic, would only require 45° of data. Comparatively, a monoclinic point group would require 180° of data around its unique axis for completeness. Therefore, the crystal symmetry can influence how many crystals are needed to obtain a complete data set. Common issues for users of the cryo-grid sample-preparation workflow are crystal loss during blotting, the observation of different thickness of the sample-embedding vitreous ice on the grid or crystal preferred orientation, creating a missing cone of data. Methods to troubleshoot and remedy these are covered in subsequent sections.

## 3. Serial crystallography

### 3.1. Overview of serial crystallography

In the purest sense, serial crystallography (SX) involves the collection of diffraction stills from randomly oriented crystals, merging multiple single exposures together to obtain a complete data set. Serial experiments can be performed at synchrotron sources (serial synchrotron crystallography, SSX) or at X-ray free-electron lasers (XFELs; serial femtosecond crystallography, SFX). A hybrid of serial and conventional methods involves the collection of multi-shot small-wedge data sets and is termed serial oscillation/rotation crystallo­graphy (SOX/SS-ROX/SF-ROX). Both SFX and SSX necessitate the collection of between 5000 and >100 000 crystal lattices, depending on the crystal system and method used, to obtain a complete data set (Mehrabi *et al.*, 2021 ▸). This extensive collection ensures high multiplicity, a critical factor during scaling to achieve optimal averaged intensities. This requirement becomes particularly important when a rocking curve is not captured and correlates with the higher *R* factors that are often observed in serial data sets. Most atomic models from serial data sets deposited in the PDB are based on 10 000–25 000 lattices.

Radiation damage remains a critical consideration in MX, SSX and SFX, with varying impacts across these techniques due to differences in exposure times, photon flux and experimental conditions (Garman & Weik, 2023 ▸). Thanks to the ‘diffraction-before-destruction’ principle enabled by the intense, ultrashort X-ray pulses (down to femtoseconds), SFX data are effectively free of radiation damage (Neutze *et al.*, 2000 ▸). However, specific radiation damage has been observed in cases where pulse lengths exceed 10 fs (Nass *et al.*, 2015 ▸; Lomb *et al.*, 2011 ▸), highlighting the importance of ultrafast exposures for achieving zero-dose conditions. In SSX, the limited photon flux necessitates much longer exposure times, making it impossible to fully avoid radiation damage. Radiation sensitivity is exacerbated by the typical room-temperature conditions of SSX experiments. Dose limits have been inferred, with a half-diffraction dose of 380 kGy for lysozyme crystals at room temperature, although site-specific damage can occur at much lower doses (∼80 kGy) and varies significantly between samples (de la Mora *et al.*, 2020 ▸; Leal *et al.*, 2013 ▸). Despite these challenges, radiation damage can be mitigated by distributing the absorbed dose over thousands of microcrystals or using low-dose exposures (Owen *et al.*, 2017 ▸). Radiation damage is less pronounced at cryogenic temperature, with a Garman limit of 30 MGy, but remains a key limiting factor in obtaining high-resolution structures (Owen *et al.*, 2006 ▸; Garman, 2010 ▸).

### 3.2. Sample delivery

**Figure 2:**
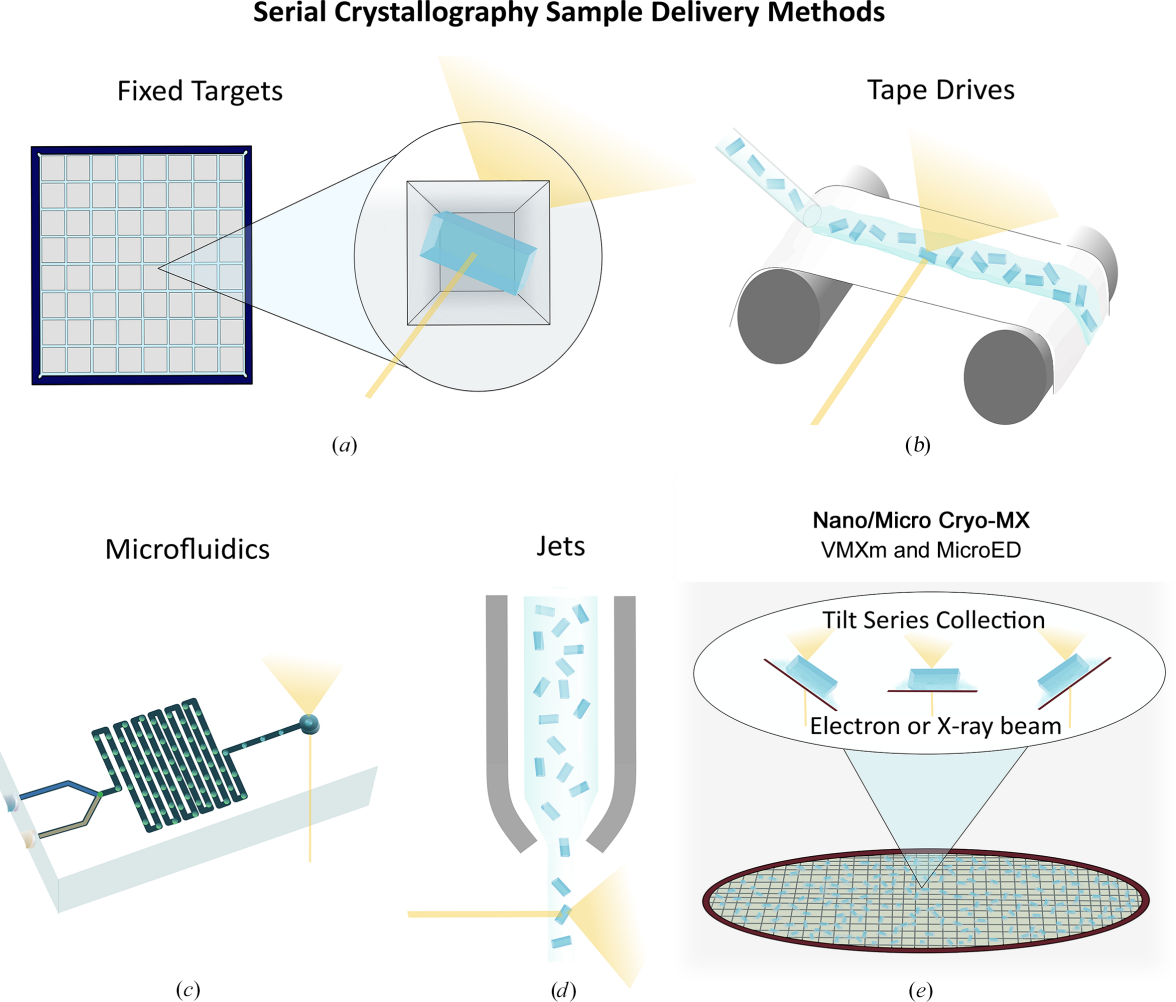
Overview of microcrystal sample-delivery methods in both serial and nano/micro cryo-MX techniques. (*a*) Fixed targets involve the immobilization of crystals in a chip with individual apertures that are rastered in a serial manner. These can be used in combination with droplet ejectors for time-resolved experiments. (*b*) Tape drives involve an X-ray-transparent tape where crystals are deposited as a stream or individual droplets. Time-resolved studies can be performed using a tape-drive strategy, with many facilities adapting systems to fit their setup. (*c*) Microfluidics can be used for *in situ* crystallization or time-resolved studies for rapid mixing experiments. (*d*) Jets provide a stream of microcrystal slurry to the beam and are the most sample-intensive method. (*e*) Nano/micro cryo-MX is an emerging field of microcrystallography currently containing MicroED and advanced beamlines such as VMXm at Diamond Light Source (Warren *et al.*, 2024 ▸). These involve the application of crystals to a carbon-coated cryoEM grid and collecting a tilt series around each crystal, making them distinct from other serial methods. Like the serial fixed targets, crystals are applied to a surface and excess solution is then blotted away to reduce background noise surrounding the sample. However, in nano/micro cryo-MX this is then vitrified to capture the crystals in amorphous ice, whereas fixed targets are collected at room temperature and crystals are captured in apertures within the solid support.

There are multiple sample-delivery modalities for serial crystallography at room temperature. The four most commonly used are jets, fixed targets, tape drives and microfluidic devices (Fig. 2 ▸ and Table 1 ▸). These approaches are now commonplace at serial beamlines; however, the exact parameters vary depending on the synchrotron or XFEL facility being used. The array of sample-delivery approaches can achieve different experimental goals; selecting the correct one for the sample and experimental aim can be challenging. An extensive comparison of serial sample-delivery methods has previously been presented (Grünbein & Kovacs, 2019 ▸; Barends *et al.*, 2022 ▸).

#### 3.2.1. Jets

Liquid jets are usually generated by a gas dynamic virtual nozzle (GDVN) through compression of a central stream of aqueous microcrystal slurry by a sheath gas flow (DePonte *et al.*, 2008 ▸). A small-diameter jet (typically 5 µm) ensures reduced background scatter and improved signal to noise, whilst the accompanying gas flow ensures that the jet deviation angle is minimal, ensuring that ice does not form and consequently plug the nozzle (Gañán-Calvo *et al.*, 2010 ▸). GDVNs are usually operated at flow rates of ∼10–60 µl min^−1^ (typical jet speeds of 10–50 m s^−1^; Schlichting, 2015 ▸). The subsequently developed double-flow focusing nozzle (DFFN) offers flow rates as low as 5 µl min^−1^, reducing the demand on sample. DFFN involves further focusing of the microcrystal slurry by an additional co-flowing solvent, resulting in a much finer liquid jet, eightfold lowered sample consumption, a further reduction in background scattering and improved reliability compared with the initial GDVN designs (Oberthuer *et al.*, 2017 ▸). However, manual fabrication of GDVNs is time-consuming and suffers inherent variability. 3D printing, based on two-photon polymerization, can reduce fabrication issues and increase the possible spatial resolution (Nelson *et al.*, 2016 ▸). Clogging in the GDVN is a potential failure point often mitigated by prior filtering of crystal size or limiting of the maximum crystal dimension to half the size of the nozzle internal diameter, ensuring a stable jet for prolonged periods.

Where the microcrystal slurry contains glycerol or PEG, the microfluidic electrokinetic sample holder (MESH) can be used as an alternative sample injector. MESH employs an electric field to generate thin jets by electrospinning at a flow rate of 0.14–3.1 µl min^−1^, with the presence of glycerol/PEG extending the microjet length, delaying droplet formation and therefore stabilizing the focused liquid stream for longer durations (Sierra *et al.*, 2012 ▸). The requirement for glycerol or PEG in the condition was addressed through the development of the concentric-flow electrokinetic holder (coMESH) injector, where microcrystals are injected into a sheath liquid such as 2-methyl-2,4-pentanediol (MPD) for electrofocusing at a flow rate of 1–3 µl min^−1^ (Sierra *et al.*, 2016 ▸).

Structural determination of membrane proteins is often performed using a high-viscosity extruder (HVE), utilizing a ∼50–100 µm stream at typical flow rates of 1–300 nl min^−1^, thus providing a sample-efficient delivery system. Crystals are grown directly in or embedded within a high-viscosity carrier matrix, the choice of which depends on multiple factors including viscosity, flow properties, compatibility with the crystal system and background scattering. The initial injector design developed at Arizona State University involved the direct injection of membrane-protein crystals grown in lipidic cubic phase (LCP) into the X-ray beam (Weierstall *et al.*, 2014 ▸). Other carriers were subsquently introduced, including mineral-oil-based greases and hydrogels such as agarose, hydroxyethyl cellulose and high-molecular-weight polyethylene oxide (Botha *et al.*, 2015 ▸). The slow flow rates are well suited to synchrotron experiments and ensure that the exposed crystal is relatively still during the long exposure time (Weinert *et al.*, 2017 ▸; Nogly *et al.*, 2015 ▸; Martin-Garcia *et al.*, 2017 ▸). The HVE approach can also be applied to soluble proteins, however further factors should be considered: the effect of shear forces (produced from mechanical mixing of protein and high viscosity carrier matrix) on crystal stability, the compatibility of the crystallization condition with the high viscosity carrier matrix of choice and the crystal density, which directly correlates to hit rate (Fromme *et al.*, 2015 ▸).

#### 3.2.2. Fixed targets

Fixed targets involve immobilization of crystals onto a solid support and subsequent scanning through the X-ray beam (Fig. 2 ▸). Hydrated crystals on fixed targets are protected with a Mylar film or kept within a humidity-controlled environment to prevent drying out (Roedig *et al.*, 2017 ▸; Oghbaey *et al.*, 2016 ▸; Mueller *et al.*, 2015 ▸; Doak *et al.*, 2024 ▸; Owen *et al.*, 2017 ▸). Depending on the fixed target used, two different methods of data collection are possible: aperture-aligned collection is used for crystals with defined locations and directed-raster methods are used where crystals are randomly distributed (Carrillo *et al.*, 2023 ▸). For fixed-target approaches, a volume of microcrystal slurry (∼100–200 µl) is pipetted onto a regular array of defined apertures (the smallest aperture currently available is 5 µm; Carrillo *et al.*, 2023 ▸). Excess liquid is removed by applying a weak vacuum to the back of the chip. In the case of the Roadrunner setup, however, filter paper is used to back-blot the chip, analogous to MicroED grid preparation (Roedig *et al.*, 2017 ▸). The chip is aligned on the beamline using imprinted fiducials, allowing efficient data collection utilizing translation stages (Sherrell *et al.*, 2015 ▸). A sealed fixed target also enables anaerobic serial data collection, when sample loading is performed in an anaerobic environment, enabling the observation of oxygen-dependent reactions within crystals (Rabe *et al.*, 2020 ▸). It is also possible to use a drop-on-demand strategy to load chips with picolitre volumes of crystal slurry at each well position without the need to pull a vacuum (Davy *et al.*, 2019 ▸).

Commonly used fixed targets for aperture-aligned data collection include the Oxford (Horrell *et al.*, 2021 ▸), HARE (Mehrabi *et al.*, 2020 ▸), MISP (Carrillo *et al.*, 2023 ▸) and Roadrunner (Roedig *et al.*, 2017 ▸) chips. The Oxford, HARE and Roadrunner chips are silicon nitride, whilst the MISP chip uses cyclic olefin copolymer (COC). Polymer approaches are now becoming the focus of fixed-target fabrication due to the cost-effectiveness and compatibility of materials such as Kapton (Bosman *et al.*, 2024 ▸). Chip design and layout vary between the three chips, with key parameters including fiducial spacing, aperture pitch and number of apertures (Owen *et al.*, 2023 ▸).

Crystal density in the slurry must be matched to the desired density of crystals per aperture: ideally one or two, or zero to one if substrates are to be added. If the crystal density is well optimized and the crystals do not show preferred orientations, two chips are generally required to obtain a complete data set. Crystals that are significantly elongated in one or two dimensions can more commonly sit in the apertures in one orientation, and in such cases more chips will be required. However, it is possible to overcome this issue by growing crystals directly on the chip through vapour diffusion (Norton-Baker *et al.*, 2021 ▸; Lieske *et al.*, 2019 ▸).

Aperture-less fixed targets involve crystal immobilization between two Mylar films held in a solid support that is directly rastered. Easy to prepare, sparing on sample (a minimum of 3 µl of crystal slurry) and comparatively quick to image (∼10 min per chip), they rarely show preferred crystal orientations and allow the collection of complete data from a single chip with an appropriate crystal density. The dominant designs are goniometer-based microgrids/micromeshes, where *MeshAndCollect* data collection is standard (Zarrine-Afsar *et al.*, 2012 ▸; Hirata *et al.*, 2014 ▸; Cohen *et al.*, 2014 ▸; Baxter *et al.*, 2016 ▸; Zander *et al.*, 2015 ▸), and Mylar sheet-on-sheet sandwiches (SOS/SOSOS chips; Doak *et al.*, 2024 ▸). Sometimes up to 100 000 images may be required as the hit rate is usually much lower than for aperture-aligned fixed targets due to the random crystal distribution. When performing a raster scan it is necessary to choose an appropriate raster spacing to avoid potential radiation-damage effects, dehydration or fouling of the film support that eventually impact data quality (Doak *et al.*, 2024 ▸).

#### 3.2.3. Tape drives

Tape drives combine the advantages of fixed targets and on-demand delivery strategies of microcrystal slurries by using a thin X-ray-transparent tape that transfers the sample into the X-ray beam. The tape material is commonly Kapton (Fig. 2 ▸). The delivery onto tape can be in the form of a train of discrete droplets using synchronized acoustic droplet ejection (ADE; typically 2–3 nl per drop), piezoelectric droplet ejection (PDE; 50–200 pl per drop) (Roessler *et al.*, 2016 ▸; Fuller *et al.*, 2017 ▸; Butryn *et al.*, 2021 ▸) or a continuous stream using a capillary or an inkjet printing nozzle (Beyerlein *et al.*, 2017 ▸; Zielinski *et al.*, 2022 ▸). In the case of on-demand ADE and/or PDE strategies, it is possible to match droplet-injection rates with the X-ray source and/or detector repetition rates to reduce sample consumption and increase hit rates, making the method amenable to both synchrotron and XFEL facilities.

Due to the comparative simplicity of this arrangement, many XFEL and synchrotron beamlines have designed bespoke systems that are compatible with their instrument, each having unique advantages and disadvantages. Primary advantages of tape drives include one-dimensional synchronization of the droplet delivery into the X-ray interaction region (*i.e.* time, via tape velocity) compared with a four-dimensional synchronization search for on-the-fly droplet delivery [droplet position (*x*, *y*, *z*) plus time]. The tape drive also provides space to initiate reactions with different strategies that are offset from the interaction region and consequently allows a wide range of reaction time points (approximately microseconds or less to tens of seconds) to be probed, which is dictated by tape velocity and distance. When everything is optimized, tape-drive methods can be among the highest throughput and most sample-efficient for serial data collection. Tape-drive methods have also been exploited for time-resolved SFX and time-resolved X-ray emission spectroscopy from the same sample and X-ray pulse to provide correlated atomic and electronic information from metallo­nzymes (Kamps *et al.*, 2024 ▸).

#### 3.2.4. Microfluidics

Microfluidics allow data collection from static crystals in chambers (akin to fixed targets) or flowing crystals within a closed channel (Fig. 2 ▸), the latter of which has very specific needs in terms of materials and bonding, without which they are prone to leaking. Appreciable channel dimensions and flow rates for microfluidic devices drastically reduce sample consumption. Flow-focusing regimes align the sample to the X-ray interaction region, ensuring high hit rates (Monteiro *et al.*, 2019 ▸). In-flow serial data collection can be as simple as flowing microcrystals through a thin-walled glass capillary (Stellato *et al.*, 2014 ▸; Ghosh *et al.*, 2023 ▸) or can require microfabricated microfluidic chips with 3D-flow focusing geometries (Monteiro *et al.*, 2020 ▸). Samples within microfluidic devices have minimal exposure to the atmosphere, presenting the additional possibility of studying anaerobic samples or conditions provided that the device is manufactured with O_2_ gas-impermeable materials. High flow rates will reduce the residency time of the sample in the X-ray beam, resulting in reduced hit rates, whilst slow flow rates are hampered by clogging of the microchannels (Monteiro *et al.*, 2020 ▸). Therefore, optimal flow rates must be carefully balanced with the selected beam size, flux and detector rate during data collection.

Microcrystals in flow within a device are unlikely to show preferred orientation. Anti-settling devices will help to prevent potential clogging within the channel or sedimentation of crystals within the input syringe itself. The application of perpetual sedimentation via syringe rotation would allow continuous delivery of crystals in a suspended state, reducing crystal losses (Lane *et al.*, 2019 ▸). The issue of clogging can be addressed by utilizing microfluidic droplets in a segmented flow injector to synchronize the delivery of sample with the X-ray beam to minimize sample consumption (Echelmeier *et al.*, 2020 ▸). However, this approach still remains technically demanding and may yield low hit rates of ∼1%, requiring lengthy data collection before a complete data set is collected (Doppler *et al.*, 2022 ▸).

## 4. Optimizing the sample for the experiment

**Figure 3:**
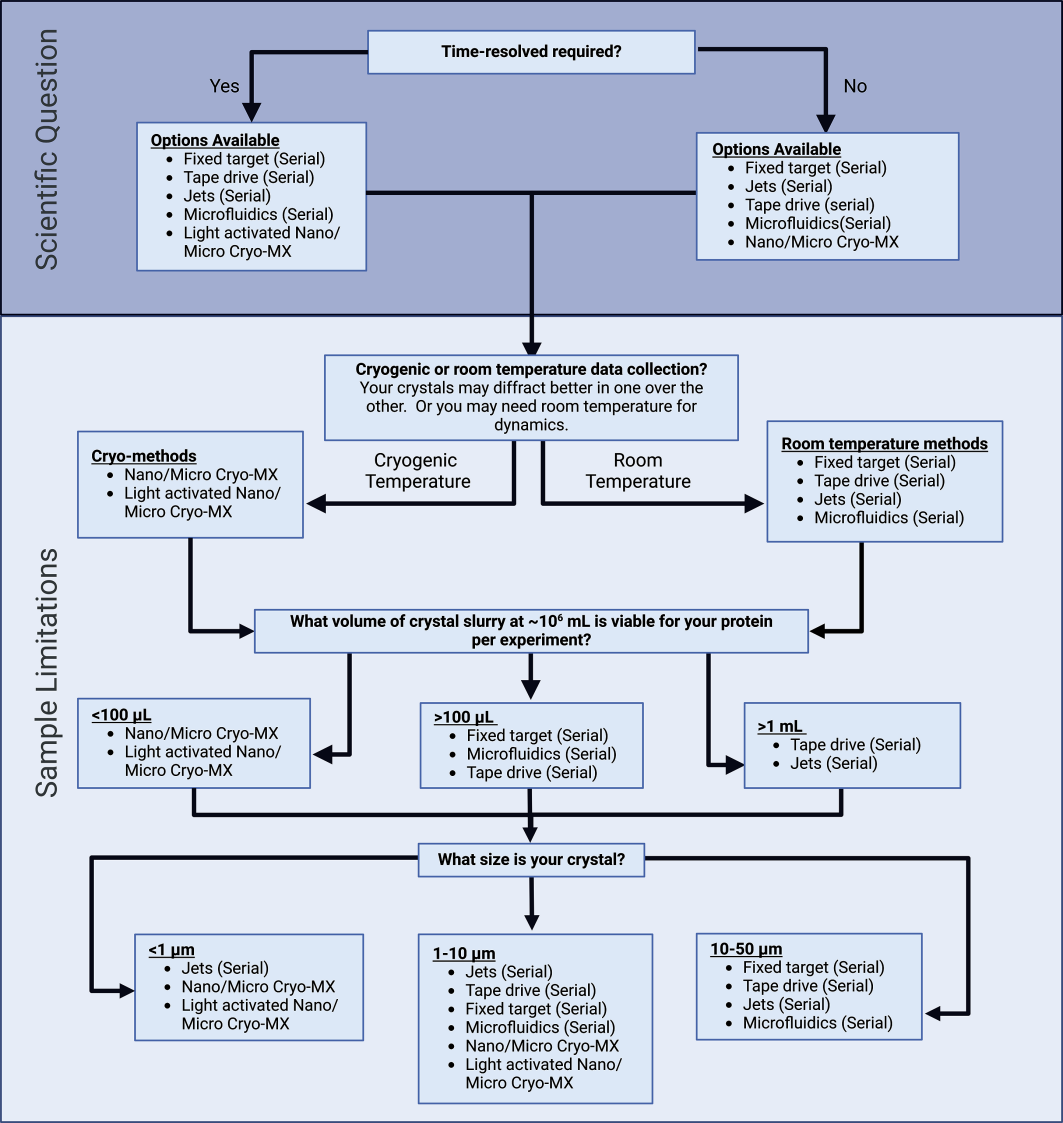
Decision flow chart to select the optimal microcrystal method for the scientific question (purple area) and the subsequent sample requirements (light blue area). This should be used as a guide for each method, taking into account the nuances of individual beamlines and equipment before attending beamtime.

As described, each method contains its own nuances of sample requirements based on the facility used, the experimental question being addressed and the sample-delivery modality chosen (Fig. 3 ▸). Therefore, the ability to tune the crystal system to obtain samples with suitable sizes, volume, density and uniformity is essential. Hence, no one singular approach will result in optimal crystals for every technique and system. The main strategies used when optimizing crystallization are phase diagrams, transitioning from vapour diffusion to batch and seeding.

### 4.1. Generating microcrystals from macrocrystals

Compared with time-resolved experiments, nano/micro cryo-MX techniques and ground-state serial structures do not require homogenous crystals. If larger crystals can be obtained, they can be crushed into suitably sized fragments (de La Cruz *et al.*, 2017 ▸). This often results in heterogeneous crystal sizes, increasing the likelihood of locating thin enough samples on cryo-grids. Additionally, this method was shown to be successful in preparing samples for tape drives, where Zielinski and coworkers vortexed 12 large urate oxidase (UOX) crystals to produce a slurry and then filtered to obtain suitable sub-20 µm crystals (Zielinski *et al.*, 2022 ▸). Although manual crystal disruption has been shown to be effective, if the parent crystal contains pathologies such as lattice defects, twinning, morphological irregularities or impurities, these will be transferred during seeding (de La Cruz *et al.*, 2017 ▸). In contrast, crystals grown to meet the size requirements may naturally contain variations between individual crystals. For most protein systems, a minimal crystal size is needed for high-resolution diffraction, and therefore protocols for fragmenting large crystals into microcrystals are essential. Large volumes of microcrystals can be produced by filtering crystals through stainless-steel filters using a HPLC pump (Shoeman *et al.*, 2023 ▸). This process achieves either ‘crystal sizing’, where large crystals are retained, or ‘crystal fragmentation’, where pressure breaks crystals apart (Shoeman *et al.*, 2023 ▸). Low crystal density and low flow rates are ideal for sizing, while high flow rates generate the pressure needed for fragmentation. The uniformity of the resulting microcrystals depends heavily on the shape and softness of the crystal (Shoeman *et al.*, 2023 ▸).

For nano/micro cryo-MX, if larger crystals can be prepared on grids, focused ion beam (FIB) milling can be used to produce suitably thin crystal lamellae (Duyvesteyn *et al.*, 2018 ▸; Zhou *et al.*, 2019 ▸; Bardin *et al.*, 2024 ▸; Noble & de Marco, 2024 ▸). This method is vastly more reproducible than manual crushing and results in an optimal crystal depth for nano/micro cryo-MX, but will inevitably be costly, labour-intensive and much lower throughput. Where large crystals cannot be manipulated physically to meet method requirements, altering crystal size by manipulating the crystallization conditions is an alternative strategy.

### 4.2. Understanding your phase diagram

**Figure 4:**
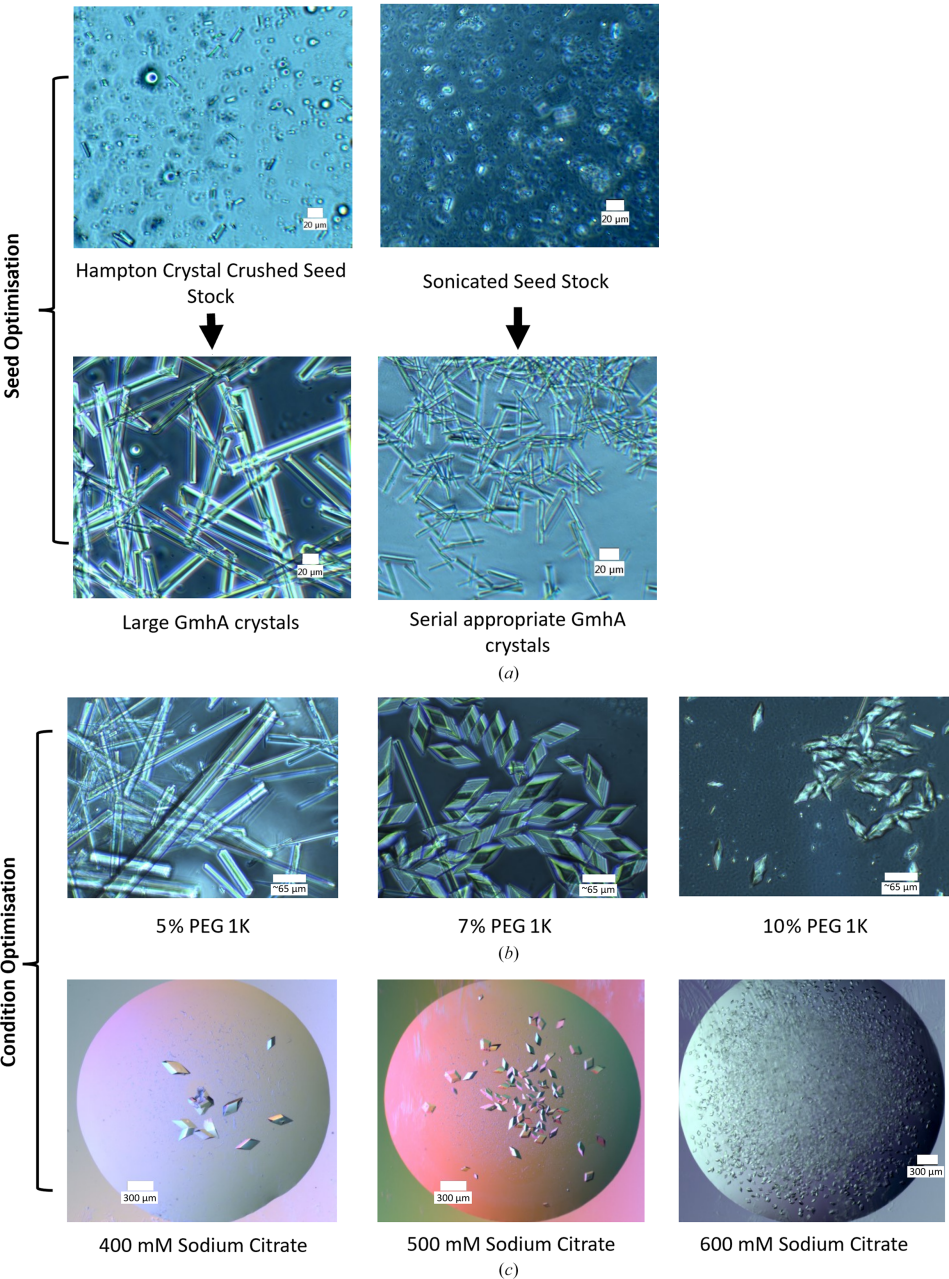
Strategies for preparing microcrystals. (*a*) Heptose isomerase GmhA protein crystals from *Burkholderia pseudomallei* showing seed optimization for size control and homogeneity. The first seed stock was made using the Hampton Research Seed Bead and was combined with mother liquor and protein, resulting in non-optimal crystals for serial data collection. The same seed stock was then sonicated for 2 min at 60% amplification (20 s on and 20 s off) to produce smaller seeds. These produced much smaller crystals with the same conditions. The scale bar is 20 µm. (*b*) Methylisocitrate lyase PrpB crystals from *Coxiella burnetii* optimized for serial data collection by increasing the concentration of PEG 1K (Stuart *et al.*, 2025 ▸). The crystals are shown to switch morphology upon increasing the PEG 1K concentration as well as reducing the crystal size. The scale bar is 65 µm. (*c*) Pdx1, an *Arabidopsis* enzyme involved in vitamin B_6_ biosynthesis, shows that an increase in salt concentration drastically reduces the crystal size and increases the number of nucleation events. The scale bar is 300 µm.

In most microcrystal projects an established crystallization condition already repeatedly yields the desired crystal form. Further fine-tuning via a combination of phase diagrams and seeding can be explored to increase the propensity of crystals with the appropriate size and distribution [Figs. 4 ▸(*b*) and 4 ▸(*c*)]. At this stage it is important to consider the final sample-delivery mode to minimize painful re-optimization later in the process. Notably, some of the modalities described are less amenable to highly viscous samples, such as nano/micro cryo-MX, jets and droplet-on-demand strategies. Therefore, options to reduce the viscosity would be preferable to minimize ice thickness, background noise or problems arising from sample delivery. Another factor is the risk of dehydration, which can occur during transit or within the crystallization condition itself, leading to crystal damage (Cheng, 2020 ▸). Tape drives and fixed targets are particularly prone to dehydration at longer time points, although some dehydration has been demonstrated to improve the crystal diffraction power (Russo Krauss *et al.*, 2012 ▸; Bowler *et al.*, 2015 ▸).

Once a condition has been decided, protein concentration and components of the mother liquor, such as pH, salt or precipitant, are changed in a stepwise fashion in either batch or vapour-diffusion crystallization experiments. Crystallization droplets are subsequently imaged, generating a visual representation and analysis of the phase diagram, allowing one to experimentally determine the undersaturated, metastable, nucleation and precipitation zones of the system. This method had been described for protein microcrystal growth before microfocus technologies had become readily available (Falkner *et al.*, 2005 ▸). By using the phase diagram, the size of the crystal and the crystal density may be tuned to meet the desired sample requirements. Additionally, the phase diagram provides a method to form a wide range of conditions to take to screening beamtime. Sometimes, crystals fail to meet the requirements for experimental data collection at this stage. To further optimize crystal density and quality, seeding methods are essential.

### 4.3. Seeding for microcrystals

Seeding, where crystalline nuclei are added to a crystallization solution to promote controlled growth in the metastable zone, was first developed in the 1980s (Thaller *et al.*, 1981 ▸). There are two forms of seeding: macroseeding and microseeding. Macroseeding uses already established single crystals as the nuclei. These are transferred to another protein solution, promoting crystal growth. Microseeding is the process of crushing previously grown crystals to form a suspension of nanocrystallites, which act as multiple nucleating points. In this section, we will be focusing on microseeding. Iterative seeding has been shown to increase crystal diffraction power and size under the original conditions (Thaller *et al.*, 1985 ▸; Dods *et al.*, 2017 ▸) and to identify new crystallization hits, termed random microseed matrix screening (rMMS; Ireton & Stoddard, 2004 ▸). This evolved into the selection of microseeds for repetitive rounds of optimization, first demonstrated by D’Arcy *et al.* (2004 ▸), to improve crystal quality as judged by X-ray diffraction.

However, there has been debate on which methods produce the ‘best’ seeds (Khurshid *et al.*, 2014 ▸; He *et al.*, 2020 ▸; Obmolova *et al.*, 2014 ▸; Castro *et al.*, 2022 ▸) and it is likely that some methods will naturally work better with specific systems. In terms of microcrystal techniques, optimum seeds should be homogeneous in their size and geometry for reproducibility, scalability, simpler data processing and consistent ligand soaking (Parambil & Heng, 2017 ▸). Seeds are almost always heterogeneous, resulting in non-uniform crystal sizes [Fig. 4 ▸(*a*)]. Variations in crystal size and non-isomorphic unit-cell distributions can lead to the exclusion of valuable experimental data. Significant variations in crystal size can cause fluctuations in measured diffraction intensities, complicating the determination of optimal exposure times (White *et al.*, 2012 ▸). These inconsistencies hinder the reliable scaling and merging of data, often resulting in the exclusion of problematic data sets (White *et al.*, 2012 ▸). Crystal size heterogeneity may also result in the presence of poorly diffracting crystals, which can affect the hit rate and indexing success, and reduce overall diffraction quality, especially if the average crystal size does not match the beam size (Darmanin *et al.*, 2016 ▸).

Each method of seeding requires the selection of the best crystals that can be sacrificed from previous successful screening (D’Arcy *et al.*, 2004 ▸). The simplest approach is to add a physical agent (for example a silicone, steel, plastic or glass bead) and vortex the Eppendorf tube vigorously, followed by a set period of time on ice. Alternatively, mechanical force can be used, crushing the crystal(s) manually with a glass probe or capillary (D’Arcy *et al.*, 2014 ▸). Sonication applies ultrasonic sound waves to the solution, causing shock waves that induce sonofragmentation of the larger crystals into seeds (Zeiger & Suslick, 2011 ▸). In doing so, the solution will continue to warm up and changes in temperature can lead to the complete dissolution of protein crystals. However, depending on the crystal system, one method may give rise to smaller homogeneous seeds than others, resulting in more consistent crystals [Fig. 4 ▸(*a*)]. Heptose isomerase GmhA from *Burkholderia pseudomallei* provides a case study of a crystal system compatible with sonication (Harmer, 2010 ▸). This protein forms rod-like crystals that are thermostable to around 90°C, making it highly resistant to heating during sonication. This was selected over mechanical force methods due to the need to form much smaller seeds than could be generated by other methods. We observed here that the larger the size of the seed, the larger the resultant crystals [Fig. 4 ▸(*a*)]. Validating the seed stock by light microscopy, TEM or dynamic light scattering (DLS) is a way to check the success of the method employed for disrupting the crystal lattices effectively (Barnes *et al.*, 2016 ▸; Stevenson *et al.*, 2014 ▸; Kadima *et al.*, 1990 ▸).

Once a seed stock has been made, it can be used at various concentrations to tune crystal nucleation, either by changing the seed concentration through phase diagrams (Fig. 4 ▸) or rMMS to obtain new conditions to optimize as above (D’Arcy *et al.*, 2014 ▸). It has been well documented that more concentrated seed stocks produce a higher concentration of smaller crystals by forming more nucleation points (Beale *et al.*, 2019 ▸). By systematically changing the protein and seed concentrations, one can often generate the desired crystal size. The optimized condition can then be scaled up as needed for particular synchrotron/XFEL applications or for nano/micro cryo-MX techniques, where the crystals can be directly applied to grids. Microcrystal techniques (especially for time-resolved studies) require multiple visits to the synchrotron or XFEL. It is therefore a requirement to produce a large stock of ‘good’ seeds, enabling reproducible methods to produce high-quality seed stock.

### 4.4. Scaling up from vapour diffusion to microbatch

For the nano/micro cryo-MX techniques, volumes up to 1 µl from batch or vapour-diffusion experiments are sufficient to transfer directly onto cryo-grids. However, if a higher crystal density or larger volumes are required, the ability to scale up a crystal condition is important. Travel to a facility can include many forms of transportation, necessitating vessels to preserve samples in transit. Serial crystallography methods can require upwards of 100 µl with a density of 10^7^–10^8^ crystals per millilitre. Scaled-up batch experiments that are easily transported to facilities in Eppendorf tubes are much more efficient for such experiments. These create a different environment to the typical crystallization plates and can lead to slight changes in the observed crystal size due to variation in evaporation conditions, altering the equilibration times between mixing and the onset of crystallization (Beale *et al.*, 2019 ▸).

The advantages of batch preparation have been outlined by Beale and coworkers, highlighting the increased control over the end result due to the removal of the transition zone between equilibration and nucleation (Beale *et al.*, 2019 ▸). Additionally, vapour-diffusion approaches are less scalable as the sample surface-area-to-volume ratio is reduced, resulting in less homogeneous crystal populations. When converting from vapour diffusion to batch, further considerations should be taken into account; precipitant and protein concentration are normally higher in vapour diffusion and equilibration occurs more slowly than in batch methods (Chayen, 1998 ▸). Precipitant concentration can be adjusted directly by increasing its concentration (typically 1.3–2 times higher in batch compared with vapour diffusion), enabling horizontal navigation of the phase diagram. Alternatively, it can be modified indirectly by varying the protein-to-precipitant ratio (commonly 1:1–1:3), resulting in a diagonal trajectory through the phase diagram. Both approaches extend the time spent in the nucleation zone, producing a greater number of small crystals and allowing fine-tuning of both crystal size and distribution (Stohrer *et al.*, 2021 ▸). Furthermore, the choice of crystallization condition will determine the rate of equilibration, as in general the more viscous PEG solutions will equilibrate more slowly than salt solutions.

In batch crystallization, rapid mixing of constituents creates a supersaturated solution, significantly enhancing nucleation. Agitating a standard batch crystallization setup, such as through microbatch mixing (MBM), has been shown to induce secondary nucleation points, facilitating the formation of microcrystals (Mahon *et al.*, 2016 ▸). However, optimizing the mixing speeds is crucial for each crystal system, as excessive agitation can compromise protein stability and reduce microcrystal yield. Rapid mixing can be combined with high-speed centrifugation to generate discrete layers of crystals, enabling the separation of crystals by size and subsequent concentration, increasing the crystal density (Tenboer *et al.*, 2014 ▸). Additionally, filtering the microcrystal slurry with appropriately sized filters allows the removal of excessively small or large crystals, ensuring a more uniform sample.

In principle, batch experiments can be scaled from nanolitre-sized droplets (in microbatch-under-oil setups) up to 200 µl batches in Eppendorf tubes. However, further investigation is required to fully understand the effect of sample volume on the resulting crystal size, density and uniformity in batch methods. With each change in scale, iterative alterations to sample ratios may be required to maintain optimal conditions.

### 4.5. Optimization of grid-based sample delivery

For nano/micro cryo-MX techniques, commonly faced sample-preparation challenges are crystal preferred orientation and ice thickness. As discussed previously, some crystals will preferentially form more surface contacts with the grid along a particular face; this is commonly associated with plate or rod morphologies. Users can either re-optimize the system to select a different crystal morphology or use alternative strategies, such as on-grid crystallization. This method involves suspending the grid in the crystallization solution, vitrifying and then using FIB milling to optimize the sample thickness (Gillman *et al.*, 2023 ▸). Not only does this allow crystals to grow in random orientations, it reduces the likelihood of sample damage during grid application.

More commonly, the sample is taken from the crystallization droplet and applied using a pipette. After blotting, the crystals must remain in a layer of amorphous ice. Smaller crystals will form less surface contact with the glow-discharged sample grids, making them susceptible to removal during sample preparation by the blotting paper (Wolff *et al.*, 2020 ▸). Using a one-sided blotter can reduce this, although crystals can still migrate to the back side of the grid and be lost in the process. A more ‘home-made’ approach with a Büchner funnel applying a vacuum to one side of the grid has been shown to increase crystal density and match the ice quality achieved by semi-automated sample-vitrification robots (Zhao *et al.*, 2021 ▸). It is crucial the ice thickness is minimal, so that background noise is minimized. Manual blotting in a humidity-controlled environment has also been used, with lower reproducibility (Nicolas *et al.*, 2024 ▸). Samples with high viscosity (for example >20% PEG) are more susceptible to thick ice as the filter paper absorption is reduced, resulting in longer blotting times, which can lead to sample dehydration. It is recommended to dilute the sample to reduce the viscosity and improve blotting (for example 5–10% PEG). Detailed grid optimization for MicroED has recently been compiled by experts in the field (Nicolas *et al.*, 2024 ▸).

### 4.6. Emerging crystallization strategies to control crystal size

Alternative approaches have been explored, including the application of microfluidics and nanotechnology. Microfluidics leverage compartmentalization for single-crystal growth and high crystal size uniformity. Droplet microfluidics have recently been harnessed, whereby the droplet acts as a controlled reaction environment, allowing the crystal size to be defined by the droplet volume. Through integration with seeding strategies, single-crystal occupancy can be achieved (Stubbs *et al.*, 2024 ▸). Similarly, complete control over the crystal system can be achieved by spatially confining the event of supersaturation to the tip of a single nanopipette (Yang *et al.*, 2023 ▸). Given the many challenges of crystallization, we can anticipate the ongoing emergence of new techniques for generating microcrystals.

## 5. Adding a fourth dimension: time-resolved approaches

The easy adoption of microcrystal strategies coincides with a paradigm shift in structural biology to observe the dynamic state(s) of macromolecules engaged in function (Orville, 2018 ▸). This is achieved, for instance, by time-resolved studies at room temperature using slurries of microcrystals, diffusing in substrates and then sampling at different time points across a reaction coordinate. These enzymatic states can then be combined into a stop-motion molecular movie with atomic spatial resolution at each time point. However, substrate transport into crystals can be a limiting factor, with traditional sized crystals (typically hundreds of micrometres in one dimension) resulting in long diffusion distances for ligands. This produces a gradient of reaction initiations across the crystal, yielding a distribution of states which significantly complicates the interpretation of electron-density maps, restricting the time points that can be studied (Schmidt, 2013 ▸). This results in overlooking earlier protein dynamics for many crystal systems. Enzymes react at approximately a factor of two slower in a crystal than in solution, as the crystal lattice limits normal-mode vibrations that are often critical to enzyme catalysis (Calvey *et al.*, 2020 ▸). Microcrystallography techniques enable us to effectively address issues related to substrate diffusion and state heterogeneity (Schmidt, 2013 ▸). It should be noted with respect to time-resolved experiments that a more viscous solution will also slow down the mixing, and ligand-diffusion times can extend collection time. Thus, further sample optimization may be necessary depending on the modality chosen.

While modern time-resolved methods emphasize room-temperature data collection, it is important not to overlook classical cryotrapping approaches, which remain effective for many protein systems when the reaction occurs on a timescale that can be captured (Caramello & Royant, 2024 ▸). Cryogenic and room-temperature structure determination each offer distinct advantages and challenges, making the choice between them highly dependent upon the experimental goal. Room-temperature data collection can offer insights under closer to native biological conditions, providing a more accurate representation of conformational ensembles, allosteric communication and ligand-binding states (Fraser *et al.*, 2011 ▸; Fischer, 2021 ▸). It enables multi-conformer refinements and avoids the need for cryoprotectants, which can alter native solvent structures (Keedy *et al.*, 2015 ▸). However, room-temperature collection is more sensitive to radiation damage, limiting tolerable doses and often requiring larger crystal volumes or multiple crystals. Maintaining crystal hydration throughout the experiment is critical, as dehydration can lead to damage and non-isomorphism. Furthermore, thermal disorder increases, contributing to higher *B* factors, diffuse background scatter and reduced Bragg intensity at large scattering angles.

Cryogenic methods, by contrast, mitigate radiation damage, allowing higher doses and extended data collection from a single crystal. Cryogenic storage and transport simplify experimental logistics. However, cryogenic methods introduce artefacts, such as the need for cryoprotectants, which can perturb solvent structures or ligand interactions (Fraser *et al.*, 2011 ▸). Irreproducible cooling can introduce variability, and ice formation can damage crystals or interfere with diffraction data.

### 5.1. 4D and serial electron diffraction

4D MicroED has been suggested for time-resolved structures from microcrystals on femtosecond timescales by Du *et al.* (2023 ▸). The vitreous environment surrounding crystals can be locally melted with a laser to trigger protein dynamics, for example with a photocaged ligand or a rapid temperature jump. The reaction can then be trapped by removing the laser, causing rapid revitrification (Lorenz, 2024 ▸). While nanojoule energy levels are required for photoactivation, a higher energy in the microjoule range will be required for melting (Voss *et al.*, 2021 ▸).

Alternative data-collection methods for nano/micro cryo-samples include serialED. Here, data are collected at specific grid positions in an automated manner, taking snapshots of crystals in random orientations and thereby eliminating the need for rotational data sets (Bücker *et al.*, 2020 ▸), a concept that evolved from serial crystallography. Currently, there are upcoming technological advancements that may lead to a convergence between MicroED and advanced microfocus beamlines (for example VMXm) with serial crystallography, such as the planned High-energy electron Xtallography Instrument (HeXI) at Diamond Light Source. Instrumentation akin to HeXI will provide both phase information and ultrafast time resolution for both large macromolecular and nanosized systems (<1 µm; Zhang *et al.*, 2021 ▸). Variations of energy may allow a greater range of sample sizes to maximize the quality of data collected (Shi & Huang, 2022 ▸) in a serial manner.

### 5.2. Time-resolved mix-and-inject serial crystallography (MISC)

A key application of serial crystallography is time-resolved experiments, for which microcrystals are ideal. The development of microfocus beamlines and the concurrent leaps in data processing have made this technique more widely accessible to non-expert users in the past few years. Experimental and data-processing workflows were first established at XFELs, which deliver short, extremely high-intensity X-ray pulses. SSX uses high-brilliance synchrotron radiation, typically with a narrow beam focus (∼1–10 µm), collecting short millisecond exposures. SSX allows the observation of biochemical processes, including conformational rearrangements, enzyme catalysis and domain motion, all of which occur on microsecond-to-second timescales (Orville, 2018 ▸).

Mix-and-inject strategies rely upon the diffusion of a substrate into the channels of a crystal. Microcrystals allow short transport paths into the crystal to allow reaction synchrony for the interrogation of millisecond-regime dynamics (Schmidt, 2013 ▸). A variety of mixing setups are available. The most common setup is a GDVN or HVE injector placed upstream of a microfluidic mixer involving a co-flow or double-focusing strategy, where mixing is dominated by diffusion (Wang *et al.*, 2014 ▸; Calvey *et al.*, 2016 ▸; Doppler *et al.*, 2022 ▸; Vakili *et al.*, 2023 ▸). This scheme is also utilized by various tape drives, where crystals are applied in a narrow stream to an X-ray-compatible tape or in discrete droplets. The distance between mixing and X-ray-interaction regions together with the time travelled defines the ligand-mixing time point (Beyerlein *et al.*, 2017 ▸; Zielinski *et al.*, 2022 ▸). Drop-on-demand approaches utilize piezoelectric droplet ejectors (PDE) to dispense picolitre-sized droplets (50–200 pl) of substrate at high frequency on top of a train of crystal-containing droplets (2–5 nl) produced by acoustic droplet ejection (ADE), also on a moving tape at a defined distance (Butryn *et al.*, 2021 ▸). This approach involves droplet impact, whereby turbulent mixing is first dominated by convection and subsequently diffusion. The PDE approach can be applied to fixed targets, whereby picolitre volumes of substrate are ejected onto crystals maintained within apertures (LAMA; Mehrabi, Schulz, Agthe *et al.*, 2019 ▸). By combining this with a Hit-And-REturn (HARE) scheme, longer timescales of the order of seconds can be accessed (Schulz *et al.*, 2018 ▸). Finally, a hybrid BITS system (comBination of Inject-and-Transfer System) can use a mixture of crystal and substrate which is injected onto a polyimide fixed target that can be raster-scanned (Lee *et al.*, 2022 ▸). Droplet microfluidics provide a strong alternative to current mixing methods, with continuous convection and diffusion inside the droplet allowing mixing to approach single-millisecond timescales (Stubbs *et al.*, 2024 ▸). However, the remaining challenge for such an approach is interfacing with the X-ray beam in a highly X-ray-transmissible device with low background noise.

### 5.3. Time-resolved pump–probe

Time-resolved strategies for light-activated systems generally involve an excitation laser pulse (pump) to illuminate the crystal, followed by an X-ray pulse (probe) to collect a diffraction pattern at a defined delay time. This allows access to a wide range of time regimes (subpicoseconds to seconds). If the system of interest is not naturally photoactivatable (only 0.5% are), an alternative is to use a photocaged substrate that is premixed with the crystals (Monteiro *et al.*, 2021 ▸). Upon light activation at a characteristic wavelength, the substrate will be released from the cage, subsequently diffusing into protein active sites. Photocages also allow perturbations such as pH jumps, a useful tool for investigating protonation states and observing intermediate states by halting reactions within pH-sensitive enzymes (Purohit *et al.*, 2024 ▸).

To ensure single-photon excitation and remove structural artefacts observed with multi-photon excitation, an appropriate pump laser fluence should be chosen, based upon a crystallographic power titration (Barends *et al.*, 2024 ▸). In addition, crystal thickness must be optimized, ensuring that it is smaller than the 1/*e* absorption depth of the pump laser at the designated wavelength of the excitation light, otherwise incomplete photoactivation may result (Grünbein *et al.*, 2020 ▸).

Liquid jets and high-viscosity extruders are commonly used to deliver photoactivatable samples, allowing a continuous laser illumination at the interaction region before interacting with X-rays in the probe region. Fixed targets, especially where crystals are maintained in a single aperture each, are well suited to laser experiments. These ensure that a single crystal is illuminated in the pump step. However, possible issues with unintended environmental pre-illumination or the accidental illumination of adjacent crystals with the pump laser should be considered (Gotthard *et al.*, 2024 ▸).

Finally, piezoelectric injectors in the drop-on-demand setup can be switched out for optical gates and lasers, allowing light-activated systems to be studied along with complementary data from X-ray emission spectroscopy (XES). This probes the redox and spin state of metals within the active site of metalloproteins (Fuller *et al.*, 2017 ▸).

## 6. Conclusions

Microcrystallography techniques hold great promise for tackling some of the most challenging questions in structural biology. By leveraging a range of crystal sizes and diverse methods for ligand delivery, researchers can tailor their approach to meet a wide variety of experimental needs. One persistent challenge, reproducibly growing crystals of adequate size and quality, is being met by strategically combining established crystallization methods. This review underscores the importance of choosing the optimal technique for each specific biological question, and it highlights the critical steps needed to optimize crystal quality for the chosen experimental setup, significantly enhancing the likelihood of success. As time-resolved studies gain momentum, there is an even greater need for refined crystal optimization to address these emerging experimental demands effectively.

## Figures and Tables

**Table 1:** Comparison of selected sample-delivery methods for serial crystallography Excluding drop-on-demand delivery directly into the X-ray beam with droplets of tens of picolitres to <5 nl in volume travelling at up to ∼4 m s^−1^ through the beam (see, for example, Roessler *et al.*, 2016 ▸; Mafuné *et al.*, 2016 ▸; Perrett *et al.*, 2024 ▸).

	Jets	Fixed targets	Tape drives	Microfluidics
Suitable crystal size (µm)	≤20 (GDVN)	∼5–50 but aperture size-dependent (aperture-aligned)	∼5–50	∼3–25
	≤50% of the internal diameter of capillary (GDVN/viscous jet)	≳1 µm (directed-raster)		
Suitable sample volume† (µl)	≥500	50–200 (aperture-aligned)	50–500	100–500
		5–10 (directed-raster)		
Sample consumption‡	Tens of milligrams or more	Tens of micrograms (directed-raster)	Hundreds to thousands of micrograms	Tens of micrograms
		Hundreds of micrograms to several milligrams depending upon sample-loading method (aperture-aligned)		
Sample-flow rate (µl min^−1^)	∼5–50 (GDVN)	N/A	∼1–10	∼1
	∼0.001–2 (viscous jet)			
Sample velocity (through the interaction region) (m s^−1^)	<200 (GDVN)	Approximately stationary	<10	<0.2
	<10 (viscous jet)			
Data-acquisition rate	<4.5 MHz (GDVN)	10–120 Hz	<500 Hz	<700 Hz
	<200 Hz (viscous jet)			
Technical difficulty	Moderate–high	Low–moderate	High	High
Compatibility with complementary data	X-ray emission spectroscopy	Electronic absorption spectroscopy	X-ray emission spectroscopy	Electronic absorption spectroscopy
			Electronic absorption spectroscopy	
Selected examples of biological case studies	Tpp49Aa1 (Williamson *et al.*, 2023 ▸)	Fluoroacetate dehalogenase (Mehrabi, Schulz, Dsouza *et al.*, 2019 ▸)	Photosystem II (Bhowmick *et al.*, 2023 ▸)	Cytochrome *c* oxidase (Ghosh *et al.*, 2023 ▸)
	CTX-M-14 (Wiedorn *et al.*, 2018 ▸)	BEV2 (Roedig *et al.*, 2017 ▸)	Urate oxidase (Zielinski *et al.*, 2022 ▸)	Aspartate α-decarboxylase (Monteiro *et al.*, 2020 ▸)
	Bacteriorhodopsin (Nogly *et al.*, 2015 ▸)	Myoglobin (Owen *et al.*, 2017 ▸)	CTX-M-15 (Butryn *et al.*, 2021 ▸)	Lysozyme (Monteiro *et al.*, 2019 ▸)

† The required sample volume is highly dependent on the crystal density.

‡ Sample consumption is assuming a data set of ≥10 000 lattices.
